# Endocrine-disrupting chemical concentrations in follicular fluid and follicular reproductive hormone levels

**DOI:** 10.1007/s10815-024-03101-0

**Published:** 2024-04-01

**Authors:** Nathalie Hoffmann-Dishon, Zohar Barnett-Itzhaki, Daniel Zalko, Rina Hemi, Nahid Farzam, Russ Hauser, Catherine Racowsky, Andrea A. Baccarelli, Ronit Machtinger

**Affiliations:** 1 Infertility and IVF Unit, Department of Obstetrics and Gynecology, Division of IVF, Sheba Medical Center, Ramat-Gan 5262000, Israel; 2 Public Health Services, Ministry of Health, 9446724 Jerusalem, Israel; 3 Faculty of Engineering, Ruppin Academic Center, 4025000 Emek Hefer, Israel; 4 Ruppin Research Group in Environmental and Social Sustainability, Ruppin Academic Center, 4025000 Emek Hefer, Israel; 5 UMR1331 Toxalim (Research Center in Food Toxicology), Université de Toulouse, INRAE, ENVT, INP-Purpan, UPS, Toulouse, France; 6 Division of Endocrinology, Diabetes and Metabolism, Sheba Medical Center, Ramat-Gan 5262000, Israel; 7 Department of Environmental Health, Harvard T.H. Chan School of Public Health, Boston, MA 02115 USA; 8 Department of Obstetrics, Gynecology and Reproductive Medicine, Hospital Foch, Suresnes, France; 9 School of Medicine, Tel-Aviv University, 6997801 Tel Aviv, Israel

**Keywords:** Follicular fluid, In vitro fertilization, Endocrine-disrupting chemicals, Reproductive hormones, Phthalates

## Abstract

**Purpose:**

To determine correlations between chemicals in follicular fluid (FF) and follicular reproductive hormone levels.

**Methods:**

The analysis was part of a larger cohort study to determine associations between exposure to EDCs and in vitro fertilization (IVF) outcomes. FF was aspirated from a single leading follicle per participant. Demographics and data on exposure to EDCs were self-reported by the participants using a questionnaire. The concentrations of estradiol (E2), progesterone (PG), anti-Mullerian hormone (AMH), and inhibin B, as well as that of 12 phthalate metabolites and 12 phenolic chemicals were measured in each FF sample. Multivariate linear regression model was used to identify the drivers of hormone levels based on participant’s age, BMI, smoking status, and chemical exposure for the monitored chemicals detected in more than 50% of the samples. Benjamini–Hochberg false discovery rate (FDR) correction was applied on the resulting *p* values (*q* value).

**Results:**

FF samples were obtained from 72 women (mean age 30.9 years). Most of the phthalates and phenolic substances monitored (21/24, 88%) were identified in FF. Ten compounds (7 phthalate metabolites, 3 phenols) were found in more than 50% of samples. In addition, there were positive associations between E2 levels and mono-n-butyl phthalate (MnBP) (beta = 0.01) and mono-isobutyl phthalate (MiBP) (beta = 0.03) levels (*q* value < 0.05).

**Conclusion:**

Higher concentrations of several phthalate metabolites, present among others in personal care products, were associated with increased E2 levels in FF. The results emphasize the need to further investigate the mechanisms of action of such EDCs on hormonal cyclicity and fertility in women.

**Supplementary Information:**

The online version contains supplementary material available at 10.1007/s10815-024-03101-0.

## Introduction

The follicular fluid (FF) microenvironment regulates oocyte quality, maturation, and subsequent embryonic development. Hormones play a crucial role in signaling and regulating folliculogenesis, enabling the development of mature oocytes [1]. For this reason, even the slightest endocrine disruption may alter the delicate hormonal balance during oocyte maturation and affect fertility.

Phthalates, as well as many phenolic substances (including bisphenols and benzophenones), are confirmed or suspected endocrine-disrupting chemicals (EDCs) present in food and everyday consumer products [2–6]. The most common sources of exposure to these chemicals include personal care products, medical devices, thermal receipts, and food packaging materials [5, 6]. Personal care products, including cosmetics, may be of particular concern for women of reproductive age. These ubiquitous compounds have been linked to adverse human health effects [7–10].

Several studies have shown a negative association between urinary levels of EDCs among women undergoing in vitro fertilization (IVF) and impaired cycle outcomes [11–15]. However, possible associations between levels of EDCs in FF and reproductive outcomes have rarely been addressed [16]. Limited research using both in vivo and in vitro models has provided evidence that some EDCs may alter the FF milieu, including alterations in the steroid profile. Changes in steroid hormone synthesis can contribute substantially to adverse reproductive effects linked with exposure to specific EDCs [17–19].

To expand on our understanding of this important area of study, in this investigation, we sought to determine correlations between chemicals in FF and follicular reproductive hormone levels.

## Materials and methods

The study was approved by our hospital’s Institutional Review Board (SMC 6140/19). All participants provided written informed consent upon enrollment.

The present study included women from a prospective preconception cohort, designed to determine associations between exposure to endocrine disruptor chemicals and IVF outcomes [13, 18]. Participants were enrolled from January 2014 to August 2016. The study recruited women aged 19 to 38 years, undergoing a first to fifth IVF treatment due to male factor, unexplained infertility, or preimplantation genetic testing for monogenic disorders (PGT-M) of autosomal recessive diseases. Patients with diminished ovarian reserve according to the Bologna criteria, endometriosis, polycystic ovary syndrome (PCOS), or those who were oocyte donors were excluded [20].

The participants’ age, body mass index (BMI), smoking status, infertility diagnosis, number of previous pregnancies and deliveries, number of IVF attempts, length of stimulation, total dose of gonadotropins, and number of oocytes retrieved and embryo quality were obtained from patients’ medical records.

All patients were treated with a gonadotropin-releasing hormone (GnRH) antagonist protocol. FF (without diluting fluid) was aspirated from a single leading follicle per participant (diameter 17–20 mm) during oocyte retrieval in one IVF cycle. In order to exclude possible confounders associated with oocyte maturation status, only FF from follicles that contained MII oocytes were included in the analysis. Day 3 top quality embryos was defined as those with 7–8 equal cells and < 10% of fragmentations [21, 22].

Following collection of the oocyte, the FF was centrifuged at 500 g for 15 min. at 4 °C for separation of cellular from the FF. The FF supernatants were collected and kept at − 80 °C until the hormonal analysis.

The concentration of four reproductive hormones, estradiol (E2), progesterone (PG), anti- Mullerian hormone (AMH), and inhibin B, were measured in the FF samples.

Estradiol was measured using a solid-phase, competitive chemiluminescent enzyme immunoassay kit (ADVIA Centaur XP, Siemens Healthcare Diagnostics, Inc. Tarrytown, NY, USA). The assay’s functional sensitivity is 69.8 pmol/L, and the intra- and inter-assay coefficient of variation (CV) ranges are 2.3–11.1% and 3.0–13.3%, respectively.

Progesterone was measured using a solid-phase, competitive chemiluminescent enzyme immunoassay kit (Immulite 2000, Siemens Healthcare Diagnostics Products Ltd., United Kingdom). The assay’s functional sensitivity is 0.64 nmol/L, and the intra- and inter-assay CV ranges are 7–17.4% and 9.5–21.7%, respectively.

AMH and inhibin B concentrations were measured using AMH Gen II enzyme-linked immunosorbent assay (ELISA) and inhibin B Gen II ELISA kits (Beckman Coulter, Fullerton, CA, USA) according to the manufacturer’s instructions. For AMH, the assay range (standard curve) is 0.16–22.5 ng/ml, and the inter- and intra-assay CV were ≤ 10.8% and ≤ 10.3%, respectively. For inhibin B, the assay range (standard curve) is 10–1000 pg/ml, and the inter- and intra-assay CV were ≤ 6.6% and ≤ 5.6%, respectively.

FF samples were shipped to the National Science Foundation (NSF, Ann Arbor, MI, USA) on dry ice. The concentrations of 12 phthalate metabolites were measured: (1) mono-benzyl phthalate (MBzP); (2) mono (3-carboxypropyl) phthalate (MCPP); (3) mono-(2-ethyl-5-carboxypentyl) phthalate (mECPP); di-(2-ethylhexyl) phthalate (DEHP) metabolites: (4) (2-ethylhexyl) phthalate (MEHP) and (5) mono (2-ethyl-5-hydroxyhexyl) phthalate (MEHHP); (6) mono (2-ethyl-5-oxohexyl) phthalate (MEOHP); (7) monoethyl phthalate (MEP); (8) mono-isobutyl phthalate (MiBP); (9) mono-isononyl phthalate (MiNP); (10) mono-n-butyl phthalate (MnBP); (11) monocarboxyoctyl phthalate (MCOMHP); and (12) monocarboxy-isononyl phthalate (MCOMOP).

The levels of 12 phenolic substances were also measured in the FF: (1) 2,4-dichlorophenol (DCP24); (2) 2,5-dichlorophenol (DCP25); (3) benzophenone-3 (BP-3); (4) bisphenol F (BPF); (5) bisphenol A (BPA); (6) bisphenol S (BPS); (7) butyl paraben (BPB); (8) ethyl paraben (EPB); (9) methyl paraben (MPB); (10) propyl paraben (PPB); (11) triclocarban (TCC); and (12) triclosan.

The NSF developed the analytical strategy based on that of the Centers for Disease Control and Prevention (CDC). The analysis was performed by solid-phase extraction in conjunction with high-performance liquid chromatography-isotope dilution tandem mass spectrometry and adhered to accepted quality assurance/quality control practices. Concentrations below the limit of detection (LOD) were assessed using instrumental reading values.

### Statistical analysis

To analyze the associations between chemicals and hormones, we used multivariate linear regressions to predict the hormone levels based on each chemical, adjusting for the participants’ age, BMI, and smoking status. Each model separately examined one chemical (out of ten chemicals whose concentration was above LOD (limit of detection) in > 50% of the samples) to one hormone (out of four hormones). Benjamini–Hochberg false discovery rate (FDR) correction was applied (Benjamini and Hochberg, 1995) to correct multi-hypotheses. Statistical significance was set at a *p* value < 0.05. For multiple comparisons, *q* value < 0.05 was set.

## Results

We analyzed FF samples from 72 women with a mean age of 30.9 ± 3.5 years and a mean BMI of 23.1 ± 4.4 kg/m^2^. About two-thirds of the participants (63.8%) underwent their first IVF cycle at the time of FF collection. Thirty women underwent PGT-M. Participant demographic and clinical characteristics are detailed in Table 1.

**Table 1:** Demographic and clinical characteristics of the study population

Variable	Participants *n* = 72 Mean ± standard deviation (range)
Age (years)	30. 9 ± 3.6 (19.3–37.6)
Body mass index (kg/m^2^)	23.1 ± 4.4 (16.3–37.3)
Gravidity (number of pregnancies)	0.9 ± 1.1 (0–5)
Parity (number of deliveries)	0.5 ± 0.7 (0–3)
Number of in vitro fertilization attempts (*n*)	1.6 ± 0.9 (1–5)
FSH dose (IU)	1740 ± 743 (750–5250)
Days of stimulation (*n*)	9.8 ± 2.0 (6–16)
Oocytes retrieved (*n*)	9.2 ± 5.3 (2–27)
Years of education (years)	15.4 ± 2.78 (12–23)

Ten chemicals were detected in at least 50% of the samples (Table 2), including three phenolic substances: one benzophenone and two parabens (BP3; MPB and PPB) and seven phthalate metabolites (MBzP, MCOMHP, MCOMOP, mECPP, MEP, MiBP, and MnBP).

**Table 2:** Chemical detection rate (µg/l) of phenols and phthalate metabolites in FF

Chemical	LOD (µg/l)	Number of samples > LOD (%) in the study population	Median (range [min–max])
DCP24	0.2	2 (3%)	0.06 (0.001–0.4)
DCP25	0.2	10 (14%)	0.04 (0.00–1.38)
BP3	0.4	38 (53%)	0.42 (0.15–12.45)
BPA	0.4	4 (6%)	0.21 (0.00–0.56)
BPF	0.4	2 (3%)	0.00 (0.00–1.81)
BPS	0.4	0	0.04 (0.00–0.25)
BPB	0.2	28 (39%)	0.15 (0.00–2.83)
EPB	1	26 (36%)	0.64 (0.15–8.45)
MPB	1	61 (85%)	2.92 (0.29–73.24)
PPB	0.2	39 (54%)	0.22 (0.00–13.85)
TCC	2	0	0.00 (0.00–0.78)
Triclosan	2	20 (28%)	0.46 (0.18–33.08)
MBzP	0.2	66 (92%)	0.52 (0.08–5.39)
MCOMHP	0.2	70 (97%)	0.54 (0.14–6.76)
MCOMOP	0.2	60 (83%)	0.26 (0.08–0.97)
MCPP	0.2	2 (3%)	0.00 (0.00–0.63)
mECPP	0.2	69 (96%)	0.49 (0.02–2.59)
MEHHP	0.1	27 (38%)	0.08 (0.00–0.34)
MEHP	1	25 (35%)	0.77 (0.01–46.82)
MEOHP	0.1	9 (13%)	0.04 (0.00–0.5)
MEP	1	53 (74%)	1.62 (0.07–22.98)
MiBP	0.2	61 (85%)	0.95 (0.01–75.25)
mlNP	0.5	0	0.08 (0.00–0.29)
MnBP	0.5	67 (93%)	1.72 (0.18–344.01)

The mean ± SD (range) FF concentrations were 2.2 ± 1.1 µmol/L (range 0–5.3) and 32.2 ± 10.8 µmol/l (range 5.9–55.0) for estradiol and progesterone, respectively. The mean ± SD (range) concentrations were 3.1 + 2.2 ng/ml (range 0.5–15.1) and 41.02 ± 31.4 ng/ml (range 5.01–163.8) for AMH, and Inhibin-B, respectively.

Higher MiBP and MnBP levels were statistically significantly associated with higher levels of E2. The results are described in Table 3. Supplemental Table 1 shows the non-significant associations between estradiol levels and FF chemicals. No other significant associations were found between the other FF hormone tested (progesterone, AMH and inhibin B) levels and FF chemicals tested.

**Table 3:** Statistically significant multivariate linear regression and association with estradiol levels

Predicted hormone	Intercept (*p* value)	Chemical name; beta (*p* value)	Age (*p* value)	BMI beta (*p* value)	Smoking status beta (*p* value)	Model *R*^2^ (RMSE)	Model *p* value (*q* value)
E2	2.40 (0.09)	MiBP; 0.03 (< 0.04)	–0.04 (0.32)	0.03 (0.32)	0.79 (0.01)	0.15 (0.99)	0.009 (0.018)
E2	2.44 (0.08)	MnBP; 0.01 (0.05)	–0.03 (0.41)	0.02 (0.4)	0.7 (0.02)	0.1 (1.02)	0.03 (0.03)

Further separate linear regressions were performed to test possible associations between FF hormone levels and FF chemicals in subgroups of: (1) FF from patients undergoing PGT-M vs. infertile women and FF chemical hormone levels, (2) FF from patients undergoing their first IVF cycle and FF from women undergoing their 2–5 IVF cycles, (3) FF that yielded day 3 top quality embryos vs. non top quality embryos. The results were null.

## Discussion

Out of 24 chemicals tested, 21 were detected in at least one FF sample, which included phthalate metabolites, bisphenols, parabens, and chlorophenols. The occurrence of several contaminants in FF was found to be low, notably for chlorophenols and TCC. The detection rates of BPA (6% of samples) and closely related analogs, were also low, which may be because bisphenols have relatively short half-lives in mammals, and because volunteers participating in this study fasted for several hours before sampling. Of note, 39% of the samples were still positive for BPB. Although the impact of this specific bisphenol has yet to be sufficiently documented, it was previously established that BPB possesses endocrine-disrupting properties and can impact steroidogenesis [23, 24]. Our findings demonstrate the presence of this substance in FF for a significant proportion of the studied population.

Our results indicate a positive association between two phthalate metabolites (MiBP, MnBP) and E2 levels. The associations between chemicals and follicular hormone levels might be of concern as oocytes are exposed to these chemicals during oocyte maturation, which is a critical step in oogenesis [25, 26].

In another study that examined possible correlations between phthalate metabolites and hormone levels in FF, monomethyl phthalate (MMP), which was not measured in the current study, was inversely associated with estradiol, progesterone, and testosterone levels. MBP and MEHHP were positively correlated with estradiol, progesterone, and testosterone levels [16]. In contrast with Du et al.’s study, we found no correlation between MBzP or MEP and hormone levels in FF. The difference in the results might be attributed to various statistical approaches, different study populations, and protocols. Over 67% of the women in Du et al.’s study were treated with a GnRH agonist long protocol, and 99% were non-smokers. In our study, all women were treated with a GnRH antagonist protocol, and 26.4% were smokers. In addition, while we excluded patients with polycystic ovary syndrome due to possibly different reproductive hormone levels, especially AMH, women with this syndrome were included in Du et al.’s cohort. In addition, inclusion criteria in Du et al.’s cohort consisted of FF from follicles > 18 mm. Consequently, some of the oocytes retrieved may have been immature, and therefore, their reproductive hormone profile may have been different. In contrast, our study included only mature oocytes.

Of note, in addition to our focus on significant substances of concern about potential EDC effects, our study has several strengths concerning the IVF protocol. Study participants were treated at a single division by the same IVF protocol (thereby preventing potential varied effects of ovarian stimulation on hormones in FF) and with a uniform method for FF collection following strict protocols. Moreover, only FF from follicles that contained mature oocytes were analyzed to overcome possible confounders of oocyte maturation status on reproductive hormone levels. However, it would have been interesting to understand if FF EDC levels correlated with immature or degenerate oocytes.

Our findings are constrained by the relatively small sample size (*n* = 72), which may have prevented us from finding more correlations between chemical metabolites and hormone levels. As this study was a further analysis of a larger study (*n* = 136), the number of FF aliquots left for this analysis was limited. In addition, FF was collected from fasting women, and it is possible that the levels do not accurately reflect the levels in the general population. As we supposed that infertile women might be more prone to EDC damage, we included both infertile women and fertile women undergoing PGT-M for the diagnosis of autosomal recessive disease or undergoing IVF for male factor infertility.

## Conclusions

Increased concentrations of selected phthalate metabolites were correlated with increased E2 levels in FF. The results highlight the possible effects of EDCs on folliculogenesis and oocyte development and emphasize the need for further research on the mechanism of action of EDCs on the hormonal cycle of female fertility.

### Supplementary Information

Below is the link to the electronic supplementary material.

Supplementary file1 (DOCX 15 KB)
