# Correction to: Endocrine-disrupting chemical concentrations in follicular fluid and follicular reproductive hormone levels

**DOI:** 10.1007/s10815-024-03133-6

**Published:** 2024-05-15

**Authors:** Nathalie Hoffmann-Dishon, Zohar Barnett-Itzhaki, Daniel Zalko, Rina Hemi, Nahid Farzam, Russ Hauser, Catherine Racowsky, Andrea A. Baccarelli, Ronit Machtinger

**Affiliations:** 1 Infertility and IVF Unit, Department of Obstetrics and Gynecology, Division of IVF, Sheba Medical Center, 5262000 Ramat-Gan, Israel; 2 Public Health Services, Ministry of Health, 9446724 Jerusalem, Israel; 3 Faculty of Engineering, Ruppin Academic Center, 4025000 Emek Hefer, Israel; 4 Ruppin Research Group in Environmental and Social Sustainability, Ruppin Academic Center, 4025000 Emek Hefer, Israel; 5 UMR1331 Toxalim (Research Center in Food Toxicology), Université de Toulouse, INRAE, ENVT, INP-Purpan, UPS, Toulouse, France; 6 Division of Endocrinology, Diabetes and Metabolism, Sheba Medical Center, 5262000 Ramat-Gan, Israel; 7 Department of Environmental Health, Harvard T.H. Chan School of Public Health, Boston, MA 02115 USA; 8 Department of Obstetrics, Gynecology and Reproductive Medicine, Hospital Foch, Suresnes, France; 9 School of Medicine, Tel-Aviv University, 6997801 Tel Aviv, Israel

**Correction to: Journal of Assisted Reproduction and Genetics**

10.1007/s10815-024-03101-0

In this article, the author’s name 'Zohar Barnett‑Itzhaki' was incorrectly written as 'Zohar Barnett‑Izhaki'. The typographical error originated in the accepted manuscript of this article. Unfortunately, the author failed to correct the error during proofing stage. The original article has been corrected.

