# Supplementary material for: Endocrine-disrupting chemical concentrations in follicular fluid and follicular reproductive hormone levels
Source: J Assist Reprod Genet. 2024 Apr 1;41(6):1637–42. doi: 10.1007/s10815-024-03101-0 (PMC11224170; doi:10.1007/s10815-024-03101-0)
Supplement: Supplementary file 1 — Supplementary file1 (DOCX 15 KB) [file 10815_2024_3101_MOESM1_ESM.docx]

**Supplementary material**

**Table S1. Multivariate linear regressions to predict Estradiol levels.**

| **Intercept (p-value)** | **Chemical name; beta**  **(p-value)** | **Age**  **(p-value)** | **BMI beta**  **(p-value)** | **Smoking status beta (p-value)** | **Model R^2^ (RMSE)** | **Model**  **q-value** |
| --- | --- | --- | --- | --- | --- | --- |
| 1.3 (0.4) | DCP24  -1.55 (0.4) | 0.008 (0.8) | 0.03 (0.39) | 0.58 (0.08) | 0.02 (1.12) | 0.35 |
| 2.11 (0.18) | DCP25  -0.16 (0.82) | -0.02 (0.67) | 0.02 (0.47) | 0.51 (0.13) | 0.06 (1.12) | 0.5 |
| 1.8 (0.21) | BP3  -0.05 (0.59) | -0.05 (0.89) | 0.02 (0.47) | 0.57 (0.06) | 0.02 (1.07) | 0.35 |
| 1.04 (0.44) | BPA  1.79 (0.13) | -0.003 (0.91) | 0.04 (0.22) | 0.38 (0.18) | 0.03 (1.01) | 0.35 |
| 1,38 (0.34) | BPF  0.5 (0.35) | 0.004 (0.91) | 0.03 (0.35) | 0.52 (0.08) | 0.03 (1.07) | 0.35 |
| 2.18 (0.31) | BPS  -0.83 (0.83) | -0.01 (0.8) | 0.01 (0.7) | 0.2 (0.62) | 0.02 (1.06) | 0.92 |
| 1.61 (0.26) | BPB  0.16 (0.57) | -0.002 (0.96) | 0.023 (0.44) | 0.53 (0.08) | 0.02 (1.07) | 0.35 |
| 1.68 (0.24) | EPB  -0.002 (0.98) | -0.003 (0.93) | 0.02 (0.44) | 0.56 (0.07) | 0.02 (1.07) | 0.35 |
| 1.92 (0.19) | MPB  -0.01 (0.4) | -0.01 (0.82) | 0.02 (0.45) | 0.57 (0.06) | 0.03 (1.07) | 0.35 |
| 2.13 (0.14) | PPB  -0.09 (0.18) | -0.02 (0.18) | 0.02 (0.47) | 0.6 (0.05) | 0.05 (1.06) | 0.35 |
| 1.67 (0.29) | TCC  0.17 (0.84) | -0.002 (0.97) | 0.02 (0.55) | 0.6 (0.09) | 0.07 (1.13) | 0.45 |
| 1.66 (0.23) | Triclosan  -0.03 (0.07) | 0.01 (0.74) | 0.007 (0.81) | 0.74 (0.02) | 0.07 (1.05) | 0.2 |
| 2.04 (0.15) | MBzP  0.19 (0.21) | -0.03 (0.49) | 0.03 (0.31) | 0.67 (0.23) | 0.07 (1.04) | 0.2 |
| 1.62 (0.26) | MCOMHP  -0.07 (0.66) | 0.0003 (0.99) | 0.02 (0.44) | 0.58 (0.06) | 0.02 (1.07) | 0.35 |
| 1.67 (0.25) | MCOMOP  0.03 (0.97) | -0.003 (0.93) | 0.02 (0.47) | 0.56 (0.07) | 0.02 (1.07) | 0.35 |
| 1.73 (0.22) | MCPP  -1.8 (0.23) | -0.0002 (0.99) | 0.02 (0.52) | 0.59 (0.05) | 0.04 (1.06) | 0.35 |
| 1.41 (0.33) | mECPP  0.2 (0.47) | 0.0002 (0.99) | 0.02 (0.43) | 0.6 (0.05) | 0.03 (1.07) | 0.24 |
| 1.25 (0.38) | MEHHP  3.9 (0.06) | -0.003 (0.93) | 0.02 (0.44) | 0.69 (0.02) | 0.07 (1.06) | 0.2 |
| 1.91 (0.16) | MEHP  -0.02 (0.41) | -0.02 (0.58) | 0.04 (0.23) | 0.66 (0.02) | 0.09 (1) | 0.08 |
| 1.84 (0.19) | MEOHP  -0.88 (0.61) | -0.02 (0.65) | 0.04 (0.24) | 0.69 (0.03) | 0.08 (1) | 0.08 |
| 1.42 (0.32) | MEP  0.04 (0.21) | 0.003 (0.93) | 0.02 (0.47) | 0.5 (0.09) | 0.4 (1.06) | 0.22 |
| 2.40 (0.09) | MiBP; 0.03 (<0.04) | -0.04 (0.32) | 0.03 (0.32) | 0.79 (0.01) | 0.15 (0.99) | 0.018 |
| 0.89 (0.61) | mINP  1.52 (0.44) | 0.01 (0.79) | 0.03 (0.36) | 0.3 (0.35) | 0.05 (1.03) | 0.64 |
| 2.44 (0.08) | MnBP;  0.01 (0.05) | -0.03 (0.41) | 0.02 (0.4) | 0.7 (0.02) | 0.1 (1.02) | 0.03 |
